# Antihypertensive Potential and Bioavailability of Peanut Protein Hydrolysate Derived from Partially Defatted Peanut Flour

**DOI:** 10.3390/foods15213908

**Published:** 2026-10-31

**Authors:** Seyi David Adebayo, Bruno Musetti, Edward M. Bahnson, Jianmei Yu

**Affiliations:** 1 Food and Nutritional Sciences Program, North Carolina Agricultural and Technical State University, Greensboro, NC 27411, USA; sdadebayo@aggies.ncat.edu; 2 Center for Nanotechnology in Drug Delivery, Department of Cell Biology & Physiology, University of North Carolina at Chapel Hill, Chapel Hill, NC 27599, USA; bruno_musetti@med.unc.edu (B.M.); edward_bahnson@med.unc.edu (E.M.B.)

**Keywords:** peanut protein hydrolysate (PPH), ACE-inhibitory activity, gastrointestinal digestion, secondary structure, FT-IR spectroscopy, antihypertensive peptides

## Abstract

This study evaluated the changes in angiotensin I-converting enzyme (ACE)-inhibitory activity and the secondary structure of peanut protein hydrolysate (PPH) during simulated gastrointestinal (GI) and serum digestions. The antihypertensive potential of PPH was validated using a spontaneously hypertensive rat (SHR) model. The PPH was produced by extensive Alcalase hydrolysis of peanut protein concentrate (PPC) and was digested by simulated gastric, intestinal, and sequential digestions, respectively. SDS-PAGE showed that PPH was relatively resistant to GI digestion. Higher single-enzyme concentration and longer digestion time increased the ACE inhibition of PPH, while sequential digestion did not change it significantly. In serum, the ACE inhibition of PPH persisted for at least 30 min, indicating that PPH peptides reaching the circulation may remain active for this period, but most of this activity was lost by 180 min. In SHRs, PPH at an oral dose of 1000 mg/kg body weight/day attenuated the rise in mean arterial pressure comparable to captopril. FT-IR demonstrated that sequential digestion reduced relative α-helices but increased β-sheet, β-turn, and random coil contents in PPH. Overall, PPH shows promise as a functional food or dietary supplement for blood pressure management. For hypertensive adults with 60 kg body weight, consuming PPH at a dose of 9.7 g per day may help control their blood pressure.

## 1. Introduction

Hypertension affects over one billion people worldwide and nearly 48% of US adults [1,2]. While antihypertensive drugs are effective, side effects can limit patient adherence [3]. Food protein-derived antihypertensive peptides offer a complementary dietary approach with minimal adverse effects [4]. Antihypertensive peptides exert their effects through multiple pathophysiological mechanisms to lower blood pressure [5]. One of the most studied mechanisms is inhibition of the renin-angiotensin system (RAS). These peptides lower blood pressure by inhibiting angiotensin I-converting enzyme (ACE, EC 3.4.15.1) and renin, two key regulatory enzymes in the RAS [6]. The RAS controls vascular constriction and dilation, renal sodium and fluid reabsorption, inflammation, and oxidative stress, all of which are pathological features of hypertension [7]. Food-derived antihypertensive peptides have been found to inhibit ACE more readily than renin [8,9]. To generate antihypertensive peptides, intact food proteins must be hydrolyzed into smaller peptides (2–20 amino acids) [10].

Proteolytic cleavage by proteases, whether added directly (e.g., pepsin, trypsin, Alcalase, papain, bromelain) or secreted by microorganisms during fermentation, liberates antihypertensive peptides from their parent protein [11,12]. The resulting peptide mixtures, called protein hydrolysates, offer significant practical advantages over purified peptides [10]. They are easily digestible [13], cheaper to produce from food by-products [14], yield higher quantities, and exhibit diverse bioactivities, including ACE inhibition [14]. Although purified single peptides are often more potent than hydrolysates, hydrolysates can be manufactured at a lower cost for large-scale production [15]. Importantly, some hydrolysates have demonstrated superior bioactivity compared to their purified peptide form [16,17], suggesting that the peptide diversity within hydrolysates determines overall antihypertensive potential.

Antihypertensive peptides can be derived from various protein sources, such as milk, eggs, fish, and plants [10]. Defatted peanut residue, a by-product of oil extraction, contains 34–52% protein depending on the degree of defatting [18] making it an economically viable source for peptide production. Global peanut production reached about 51.8 million metric tons in the 2024/2025 marketing year, with China being the largest producer followed by India, Nigeria and the United States [19]. Roughly 19.4 million metric tons (37.45%) were crushed for oil that year, yielding about 7.8 million metric tons of defatted meal worldwide [19]. Although predominantly used as livestock feed, this protein-rich by-product could be valorized for human consumption and value-added product development. Additionally, peanut protein is exceptionally rich in arginine, a precursor of nitric oxide (NO) synthesis, an important vasodilator that helps regulate blood pressure [20]. Arginine supplementation has demonstrated significant antihypertensive effects in both animal models and human subjects [21,22]. Peanut protein hydrolysates (PPH) have been shown to have significant ACE-inhibitory activity in vitro [23,24,25,26,27] and in vivo [28]. PPH produced via Alcalase hydrolysis generates peptides with multifunctional bioactivities, including ACE/renin inhibition and antioxidant properties [24,27]. Alcalase hydrolysis has also been reported to significantly reduce the allergenic potential of peanut flour [26,27] which could reduce the severity of allergy [29]. In addition, proper labeling can significantly reduce the incidence of peanut allergy caused by accidental exposure.

The enhanced bioactivity and reduced allergenicity position PPH as a viable functional food ingredient or dietary supplement. However, an important gap exists in understanding how gastrointestinal digestion and serum peptidases affect PPH bioactivity during absorption and metabolism. Following ingestion, proteins and peptides undergo sequential proteolysis by pepsin in the stomach, pancreatic and intestinal enzymes in the small intestine, and brush-border peptidases [30]. After absorption into the bloodstream, serum peptidases continue to hydrolyze absorbed peptides [31]. Most ACE-inhibitory peptides contain fewer than 20 amino acids [10]. Amino acid sequence, molecular weight, and hydrophobicity are the main predictors of their activity, particularly in peptides shorter than 8 amino acids (AAs) [32]. Peptides smaller than 15 AAs typically do not adopt a stable secondary structure, and short sequences rarely keep a defined fold once released from the parent protein [33]. An 8-residue ACE-inhibitory peptide purified from an Alcalase hydrolysate of seahorse protein, for example, was mainly random coil [34]. Predicted structures of the peptides in the AHTPDB antihypertensive peptide database showed the same pattern. In peptides shorter than 5 residues, all AAs were in loop regions, and in peptides of 5 to 10 residues only about 1% formed a helix or strand [35]. The three-dimensional arrangement of short peptides in the active site still affects their activity. Docking and molecular dynamics of ACE-inhibitory tripeptides showed that their potency depended on how well their side chains fitted the hydrophobic pockets and polar sites of ACE [36]. Secondary structure may also affect the activity of peptides with more than 8 amino acids by controlling the access to the binding sites of ACE [32], and predicted helices, strands and turns are more frequent in antihypertensive peptides of 11 to 30 residues [35]. Conflicting results have so far prevented a consensus on which factor drives ACE inhibition [37,38,39]. FTIR of a protein hydrolysate records the relative contents of different secondary structural elements of the peptide mixture, so it can illustrate how digestion shifts the contents of these elements in the hydrolysate. We hypothesized that these changes would relate to the ACE-inhibitory activity of the digesta. This study evaluated whether the ACE-inhibitory activity of PPH was sustained under simulated gastrointestinal (GI) digestion conditions and in the presence of serum peptidases, and tested its antihypertensive effect in spontaneously hypertensive rats (SHRs). The study also examined how GI digestion changes the secondary structure of PPC and PPH and whether these changes relate to their ACE-inhibitory activity.

## 2. Materials and Methods

### 2.1. Materials

Light-roasted partially defatted peanut flour (containing 12% lipid and 50 ± 2% protein) was purchased from the Golden Peanut Company (Albany, GA, USA). This food-grade flour is a partially defatted by-product of peanut oil production and was also the starting material of our earlier study on the optimization of Alcalase hydrolysis of peanut protein concentrate to produce highly effective ACE and renin inhibitory PPH [27]. Alcalase 2.4 L (with subtilisin from *Bacillus licheniformis* as dominating enzyme) was obtained from Novozymes (Franklinton, NC, USA). Pepsin (porcine gastric mucosa, ≥400 U/mg) and angiotensin I-converting enzyme (ACE; 0.25 U/mL, rabbit lung) were obtained from Sigma-Aldrich (St. Louis, MO, USA). Pancreatin (porcine, 8X USP, protease activity ≥ 200 U/mg) was purchased from MP Biomedicals (Irvine, CA, USA). Other analytical grade chemicals, including KH_2_PO_4_, sodium chloride, sodium hydroxide, Tris base, ammonium persulfate, and bromophenol blue, were purchased from Fisher Scientific (Waltham, MA, USA), while acrylamide, bis-acrylamide, and SDS were purchased from Bio-Rad (Hercules, CA, USA) and TEMED from RPI (Chicago, IL, USA). The Pierce BCA Protein Assay Kit, including bovine serum albumin standards, was purchased from Thermo Scientific (Rockford, IL, USA). Organic solvents were purchased as follows: ethanol from VWR BDH (Philadelphia, PA, USA), methanol from Alfa Aesar (Haverhill, MA, USA), and glycerol and β-mercaptoethanol from Acros Organics (Morris Plains, NJ, USA). Simulated gastric fluid was purchased from Ricca Chemical (Arlington, TX, USA), and simulated intestinal fluid without pancreatin was prepared in accordance with USP 26 guidelines.

### 2.2. Preparation of Peanut Protein Concentrate

Peanut protein concentrate (PPC) was prepared following a previously reported method [27]. Briefly, 100 g of partially defatted peanut flour was dispersed in 1 L of deionized water. The pH was adjusted to and maintained at 10.0 ± 0.1 using 2.0 N NaOH, and the mixture was stirred continuously at 35 °C for 60 min. After centrifugation at 3000× *g* for 20 min in an Eppendorf 5810R centrifuge (Eppendorf SE, Hamburg, Germany), the supernatant was acidified to pH 4.8 ± 0.2 with 2 N HCl and then centrifuged again under the same conditions. The precipitate was frozen at −80 °C and subsequently freeze-dried for 24 h. The dried PPC was ground into powder and stored at 4 °C in moisture-proof containers.

### 2.3. Hydrolysis of PPC to Produce Peanut Protein Hydrolysate (PPH)

Alcalase hydrolysis of PPC was conducted following a previously reported method [27]. Briefly, 100 g of PPC powder was suspended in 1 L of deionized water, adjusted to pH 8.0 ± 0.2, and equilibrated at 40 °C in a Julabo SW22 shaking water bath (Julabo GmbH, Seelbach, Baden-Württemberg, Germany). Hydrolysis was initiated by adding 4 mL of Alcalase 2.4 L, an enzyme-to-substrate ratio of 4% (*v*/*w*), and was allowed to proceed for 6 h at 40 °C and 160 rpm. Throughout hydrolysis, the pH was held at 8.0 ± 0.2 by dropwise addition of 2 N NaOH. The total volume of base used was recorded. Enzyme activity was terminated by heating at 95 °C for 10 min, followed by cooling in an ice-water bath. The hydrolysate was centrifuged at 3000× *g* for 20 min, and the supernatant was freeze-dried and stored at 4 °C.

### 2.4. In Vitro Gastrointestinal Digestion of PPC and PPH

PPC and PPH were digested in vitro under gastric and intestinal conditions using a protocol adapted from Brodkorb et al. (2019) [40]. Dry PPC and PPH were reconstituted to 10% (*w*/*v*) in either simulated gastric fluid (SGF, without pepsin; Ricca Chemical) or simulated intestinal fluid (SIF, without pancreatin; prepared according to USP 26), each pre-warmed to 37 °C. Reconstitution of the substrate shifted the pH of both fluids, so the pH was corrected before enzyme addition and then maintained throughout digestion by dropwise addition of 1N HCl (gastric) or 1N NaOH (intestinal). Pepsin and pancreatin were each added to the final concentrations of 0.5, 1.0, 1.5, and 2.0 mg/mL, with 1.0 mg/mL as the reference concentration, which falls within the range reported for pepsin in human gastric fluid [41], and the same concentrations were applied to pancreatin to allow direct comparison between the two conditions. The remaining concentrations were included to evaluate the effect of enzyme concentration on digestion. PPC was digested at the reference concentration only. For simulated gastric digestion (SGD), samples in SGF were adjusted to pH 2.0, pepsin was added, and digestion was conducted at 37 °C for 1 or 2 h while maintaining pH at 2.0. For simulated intestinal digestion (SID), samples in SIF were brought to pH 7.0, pancreatin was added, and digestion was conducted at 37 °C for 1 or 2 h with the pH maintained at 7.0. For sequential digestion (SEQ), samples in SGF were brought to pH 2.0, pepsin was added to 1.0 mg/mL, and digestion was conducted at 37 °C for 1 or 2 h with the pH held at 2.0 ± 0.1. Pepsin was then inactivated by raising the pH to 8.0 with 2N NaOH. An equal volume of pre-warmed SIF was added; the pH was then brought to 7.0, and pancreatin was added to 1.0 mg/mL. Digestion was conducted at 37 °C for a further 1 or 2 h, with the pH maintained at 7.0; sequentially digested samples received 2 or 4 h of digestion in total. In all conditions, enzyme activity was terminated by heating at 95 °C for 10 min. Samples were cooled on ice, centrifuged at 10,000× *g* for 15 min, and supernatants were stored at −20 °C for further analysis.

### 2.5. Human Serum Digestion of PPH

Human serum digestion was conducted with minor modifications to previously reported methods [42]. Frozen normal human serum (Sigma-Aldrich, St. Louis, MO, USA), pooled from multiple donors, was thawed on ice and centrifuged at 14,000× *g* for 10 min to remove lipids and particulates. The serum was not heat-inactivated, so that its native peptidase activity was preserved. The clarified serum was pre-warmed to 37 °C in a water bath. Undigested PPH and its GI digesta were combined with serum in a set of sterile tubes to a final PPH concentration of 5 mg/mL, gently mixed, and incubated at 37 °C in a shaking water bath. At 0, 10, 30, and 180 min, proteolytic activity was halted by adding 300 µL of 2 N HCl. The mixtures were centrifuged at 10,000× *g* for 2–3 min, and 1000 µL aliquots of the supernatant were stored at −20 °C until ACE-inhibitory activity measurement. The supernatants were diluted to 2 mg/mL PPH for the ACE-inhibitory assay. The serum was a commercial, de-identified product, and its use did not require institutional review board approval because the study is classified as not human subjects research according to the US federal Common Rule (45 CFR 46) [43].

### 2.6. SDS-PAGE Analysis

Protein and peptide degradation during simulated digestion was monitored by SDS-PAGE. Gradient Tris-Tricine gels (4% stacking, 10% spacer, 16% separating) were cast and polymerized according to manufacturer instructions. Peptide samples were centrifuged at 10,000× *g* for 10 min. Supernatants were diluted with phosphate buffer to 4 mg/mL and then mixed 1:1 with 2× Laemmli sample treatment buffer (pH 6.8) to a final protein concentration of 2000 µg/mL. PPC and PPH supernatant samples were loaded onto the gel at 10 µL/well and 15 µL/well, respectively. Precipitated fractions were resuspended in Laemmli buffer and similarly prepared; 5 µL was loaded per well. All samples were denatured at 95 °C for 5 min before electrophoresis. A prestained molecular weight marker (2–250 kDa) served as a reference. Electrophoresis was conducted using a Mini-PROTEAN Tetra Cell (Bio-Rad, Hercules, CA, USA). Gels were stained with Coomassie Brilliant Blue, destained, and protein and peptide bands were visualized on a UVP ChemStudio imaging system (Analytik Jena, Upland, CA, USA) to estimate molecular weights.

### 2.7. Protein Quantification Using BCA Assay

Protein content in samples digested at the reference enzyme concentration (1.0 mg/mL) was measured by the bicinchoninic Acid (BCA) microplate method using the Pierce™ BCA Protein Assay Kit purchased from Thermo Scientific. The working reagent was freshly prepared by mixing Reagent A and Reagent B in the kit at 50:1 ratio (*v*/*v*), as specified by the manufacturer. A 96-well microplate received 25 μL of protein standards, unknown samples, or buffer blank in triplicate, followed by 200 μL working reagent. Plates were covered, shaken for 30 s on a Lab-Line Model 4625 titer plate shaker (Barnstead, Melrose Park, IL, USA), incubated at 37 °C for 30 min, and cooled to room temperature. Absorbance was measured at 562 nm. Buffer blank values were subtracted, and protein concentrations were determined from a standard curve developed using bovine serum albumin (BSA) standard solutions (0.25–2 mg/mL).

### 2.8. Free Amino Acid Quantification

Free amino acids in the samples digested at the reference enzyme concentration (1.0 mg/mL) were quantified using the ninhydrin method (Sigma-Aldrich). A standard calibration curve was prepared using leucine solutions at concentrations of 12.5, 25, 37.5, 50, 100, 150, and 200 µM in 0.05% glacial acetic acid. Standard and sample solutions (2.0 mL) were mixed with 1.0 mL of the ninhydrin reagent and vortexed. A blank was prepared by mixing 2.0 mL of deionized water with 1.0 mL of ninhydrin reagent. All tubes were heated in a boiling water bath for 10 min to develop the chromogenic complex, cooled on ice, and 5.0 mL of 95% ethanol was added in each tube to stabilize the color. Absorbance was measured at 570 nm using a Genesys 10 UV spectrophotometer (Thermo Scientific, Madison, WI, USA). Blank values were subtracted, and free amino acid concentrations were calculated using the standard curve developed using leucine solutions.

### 2.9. ACE-Inhibitory Activity

ACE-inhibitory activity was assessed using a previously reported method with slight modification [27]. Undigested and GI-digested PPC and PPH samples were diluted to final concentrations of 2, 4, 6, 8, and 10 mg/mL in 50 mM Tris–HCl buffer (pH 7.5) containing 0.3 M NaCl, with the corresponding undigested samples serving as controls for the GI-digested samples. Captopril, diluted in the same buffer at 0.001, 0.005, 0.01, 0.05, and 0.1 µg/mL, served as the positive control. Human serum-digested PPH was evaluated at a fixed concentration of 2 mg/mL, which matches the lowest concentration tested for the GI digesta. This concentration lies below the IC_50_ values of undigested PPH and its digesta at the reference enzyme concentration. Inhibition is therefore not saturated at 2 mg/mL, so a gain or a loss of activity during serum incubation can be detected. To validate assay responsiveness, captopril was spiked into the serum at 5 µg/mL as a positive control. Serum-containing phosphate buffer without PPH or captopril served as a negative control. Its percentage inhibition was subtracted from that of each PPH–serum sample to correct for the contribution of the serum matrix. The substrate FAPGG was prepared at 0.4 mg/mL in 50 mM Tris-HCl buffer containing 0.3 M NaCl (pH 7.5) and pre-incubated at 37 °C. In a 96-well microplate, each well received 10 µL of ACE solution (0.25 U/mL) and 10 µL of captopril, PPC, PPH, or Tris-HCl buffer (for the uninhibited reaction), and the plate was gently shaken for 2 min on a titer plate shaker. Subsequently, 150 µL of FAPGG substrate pre-warmed to 37 °C was added to initiate the reaction. Absorbance at 340 nm was measured every minute for 30 min at 37 °C using a Synergy HTX microplate reader (BioTek Instruments, Winooski, VT, USA). ACE inhibitory activity was calculated from the linear portion of the absorbance–time curve with the formula

ACE Inhibition (%) = [1 − (S_inhibitor_/S_no inhibitor_)] × 100 (1)

where S_inhibitor_ is the slope for the peptide samples, and S_no inhibitor_ is the slope for the uninhibited reaction. For undigested and GI-digested samples and for captopril, the IC_50_ value (the concentration required to inhibit 50% of ACE activity) was determined using nonlinear regression analysis. ACE-inhibitory activity of human serum-digested PPH samples was reported as serum-corrected percentage inhibition at 2 mg/mL.

### 2.10. Fourier Transform Infrared (FT-IR) Spectroscopy and Protein Secondary Structure Determination

FT-IR absorbance spectra of PPC and PPH samples were obtained using a Nicolet™ iS50 FTIR Spectrometer equipped with a diamond Attenuated Total Reflectance (ATR) detector and Mercury Cadmium Telluride (MCT) detector (Thermo Scientific). The system was purged with nitrogen for 2 h to eliminate interference from CO_2_ and water vapor. Background spectra were collected in air, followed by buffer and sample spectra. Buffer signals were subtracted from sample spectra to isolate protein signals. Spectral acquisition involved 256 scans at 4 cm^−1^ resolution over 4000–800 cm^−1^. The protein secondary structure was determined by deconvolution of FT-IR spectra using ProtaCAL Software Suite (BioTools, Jupiter, FL, USA) to quantify α-helices, β-sheets, bends/turns, and random coils as percentages of the total protein structure.

### 2.11. Validation of Antihypertensive Potential of PPH Using a Rat Model

An in vivo study was conducted to validate the antihypertensive potential of PPH. Forty-eight (48) 10-week-old male spontaneously hypertensive rats (SHRs) were purchased from Envigo (Indianapolis, IN, USA), and they were randomly assigned into 6 groups with 8 rats in each group (*n* = 8): (1) negative control (saline), (2) positive control 1—ACE inhibitor Captopril (1 mg/kg bw/day), (3) positive control 2—renin inhibitor Aliskiren (1 mg/kg bw/day), (4) PPC (1000 mg/kg bw/day), (5) low-dose PPH (250 mg/kg bw/day), and (6) high-dose PPH (1000 mg/kg bw/day). Power analysis was conducted using G*Power (version 3.1) before the study according to a published SHR study with a peanut-derived product, in which the smallest reduction of about 10 mmHg in systolic blood pressure (SBP) with 6.65 mmHg standard deviation of SBP measurement was reported [44]. Detecting this difference at a significance level of 0.05 with 80% power required 7.94 rats per group [45], so eight rats were used in each treatment group. The doses were based on earlier SHR studies of peanut-derived products, in which an extract of fermented peanut meal lowered SBP at 200 and 500 mg/kg bw [44], and a peanut protein-derived peptide was given at 100 to 1000 mg/kg bw/day for 28 days [46]. Aliskiren, a direct renin inhibitor, was included as a second positive control because our previous study demonstrated the renin-inhibitory activity of PPH [27]. The rats were tagged and pair-housed in the cages at constant room temperature (23 ± 0.5 °C) and humidity (55%), with a 12-h light–dark cycle. After one week of acclimation, their baseline mean arterial blood pressure (MAP) was recorded, and treatments were given by daily oral gavage for 8 weeks. The MAP was measured 4 h post-gavage weekly using a non-invasive tail-cuff method with a Volume Pressure Recording System (4-rat CODA, Kent Scientific Corporation, Torrington, CT, USA). Mean arterial pressure (MAP) was automatically measured and recorded by the CODA system.

This time point was chosen because earlier SHR studies of peanut-derived products found the largest blood pressure reduction 3 to 4 h after oral administration [44,46]. Tail-cuff measurement requires restraint and warming, which can raise blood pressure, so repeated measurements at several times after each dose were not made, as they would have added stress and could have masked the treatment effect. Before the MAP measurement, the rats were kept on a warming pad at 30 °C for 5 min to make tail artery pulsations detectable. Throughout the experiment, all rats had free access to water and feed (AIN-93M). The animal study was completed according to a protocol (19–220.0-C) approved by the Institutional Animal Care and Use Committee (IACUC) at the University of North Carolina at Chapel Hill.

### 2.12. Data Analysis

Raw data from protein assays, free amino acid quantification, and ACE inhibition tests were expressed as mean ± standard deviation and statistically analyzed using GraphPad Prism version 11.1.0. The IC_50_ values for ACE-inhibitory activity were determined by nonlinear regression. Normality of residuals was checked with the Shapiro–Wilk test, and equality of variances with the Brown–Forsythe test for one-way designs and Spearman’s test for heteroscedasticity for two-way designs, before parametric analysis. The IC_50_ data, the serum ACE inhibition data, and the FAA and protein/peptide contents of PPH met both assumptions. The FAA contents of PPC met them after log_10_ transformation and were analyzed on that scale, but they are shown as untransformed means ± SD. The protein/peptide contents of PPC deviated slightly from normality (Shapiro–Wilk, *p* = 0.035) but had homogeneous variances (Brown–Forsythe, *p* = 0.46), and one-way ANOVA was kept for these data, as the F test is robust to moderate departures from normality [47]. Protein and free amino acid concentrations were compared using one-way ANOVA followed by a post-hoc Tukey test (*p* < 0.05). For PPC, the effects of enzyme type and digestion time on IC_50_ were analyzed by two-way ANOVA. For pepsin- and pancreatin-digested PPH, the effects of enzyme concentration and digestion time on the IC_50_ of PPH were analyzed by two-way ANOVA. The IC_50_ values within same substrate (PPC or PPH) were compared using a post-hoc Tukey test (*p* < 0.05). ACE inhibition of serum-incubated samples was analyzed by two-way ANOVA (sample × incubation time) followed by Tukey’s test, which compared the incubation times within each sample. Post-hoc Tukey’s multiple comparison was performed to compare the MAP values of SHRs in different treatment groups at same treatment time (week) at 5% significance level. Secondary-structure contents are presented as mean ± SD without significance testing, because most of these datasets did not meet the normality assumption. A Pearson correlation matrix was constructed for the relationship between the relative content of a specific secondary structure and ACE-inhibitory activity (IC_50_), and a two-tailed *p*-value was calculated for each coefficient (*n* = 7 samples per substrate).

## 3. Results and Discussion

### 3.1. Effect of Simulated GI Digestion on Molecular Weight Distribution of PPC and PPH

**Figure 1:**
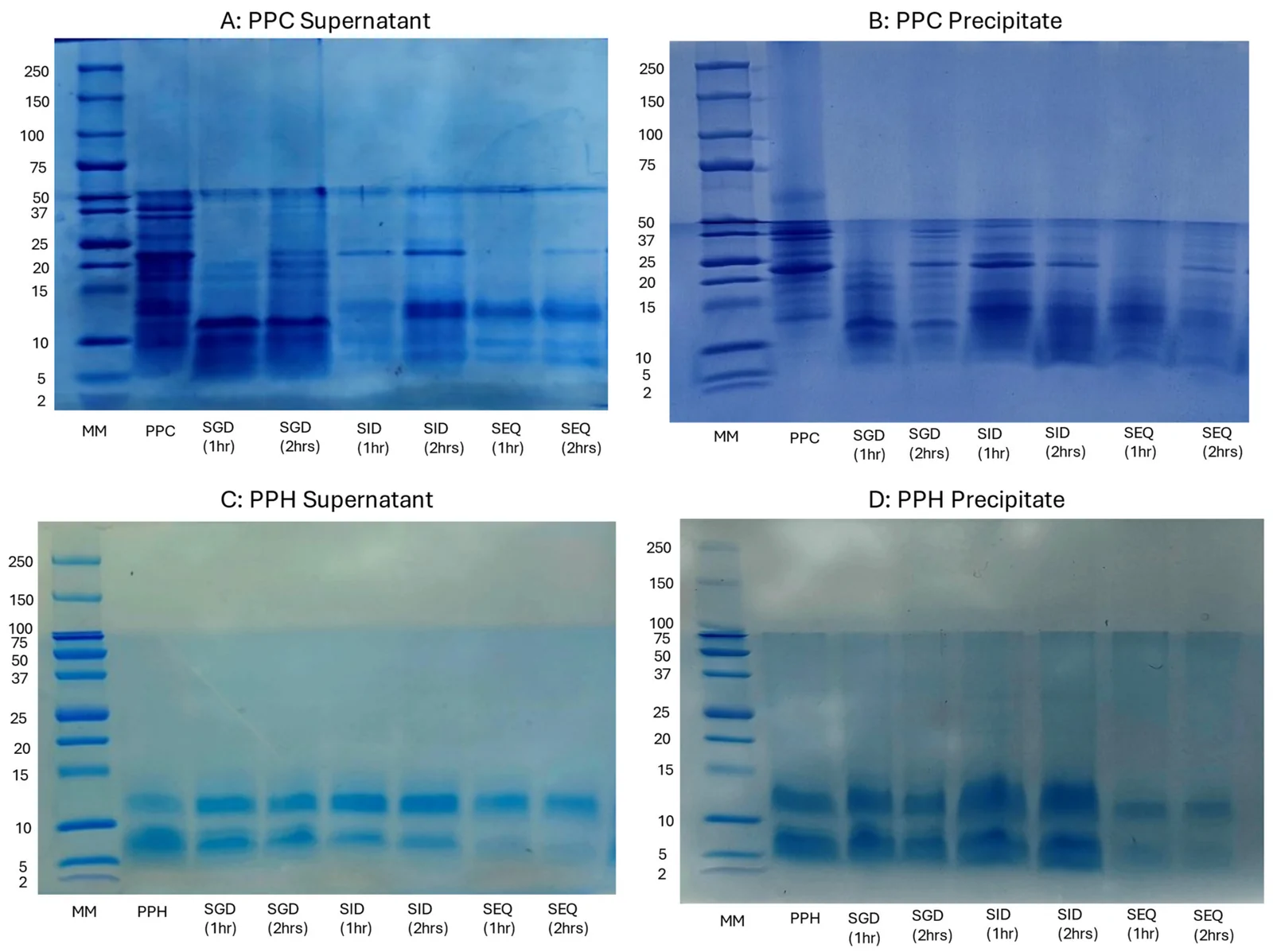
SDS-PAGEs of soluble (supernatant) and insoluble (precipitate) proteins/peptides from PPC (**A**,**B**) and PPH (**C**,**D**) digested for 1 or 2 h under simulated gastric (SGD), intestinal (SID), or sequential (SEQ) digestion conditions. MM: molecular marker.

Undigested PPC had a protein profile with nine distinct bands with molecular weights (MW) 7.98 to 50 kDa in the supernatant (Figure 1A) and nine bands in the precipitate with MW 13.58 to 64.74 kDa (Figure 1B). The 64.74 kDa band, which likely corresponds to Ara h1 based on peanut allergen database [48], was absent across all digested samples, consistent with the known susceptibility of Ara h1 to gastrointestinal digestion [49]. Among single-phase treatments, SGD with pepsin produced modest changes in PPC profile (Figure 1A,B). SID with pancreatin reduced the number of bands originally present in PPC and generated lower-molecular-weight peptides in both soluble and insoluble fractions (Figure 1A,B). Band intensity in the precipitate decreased with extended digestion time, suggesting continued peptide breakdown. Notably, pepsin produced predominantly water-soluble peptides that were enriched in the supernatant, whereas pancreatin retained more insoluble peptides in the precipitate, suggesting that the two enzymes generate peptides with distinct solubility characteristics. Sequential digestion achieved the most extensive hydrolysis of PPC, reducing the protein profile to four bands in the supernatant after 1 h, with an additional band at approximately 4.59 kDa after 2 h (Figure 1A), while the intensity of the precipitate bands decreased progressively (Figure 1B).

PPH control had only two primary bands at approximately 11 kDa and 7.7 kDa in the supernatant (Figure 1C) and three bands with MW around 11 kDa, 6.04 kDa, and 5.36 kDa in the precipitate (Figure 1D), confirming that Alcalase pre-hydrolysis had already reduced intact proteins to low-molecular-weight peptides. Peptides smaller than 5.36 kDa may have run off the gel and could not be detected by the 16% Tris-tricine gel. Among single-phase treatments, SGD with pepsin had negligible effects on the number and intensity of bands in either fraction (Figure 1C,D). SID with pancreatin reduced the band intensity of the 7.7 kDa peptide in the supernatant (Figure 1C) but increased the intensities of all peptide bands in the precipitate (Figure 1D), suggesting the accumulation of hydrophobic peptides resistant to pancreatin hydrolysis. Sequential digestion reduced peptide band intensity in both fractions (Figure 1C,D), particularly at the 5.36 and 6.04 kDa bands, but did not eliminate these peptides, confirming that even combined pepsin and pancreatin action could not fully degrade all Alcalase-generated peptides.

### 3.2. Effect of Simulated GI Digestion on Protein/Peptide Content and Free Amino Acid Release

**Figure 2:**
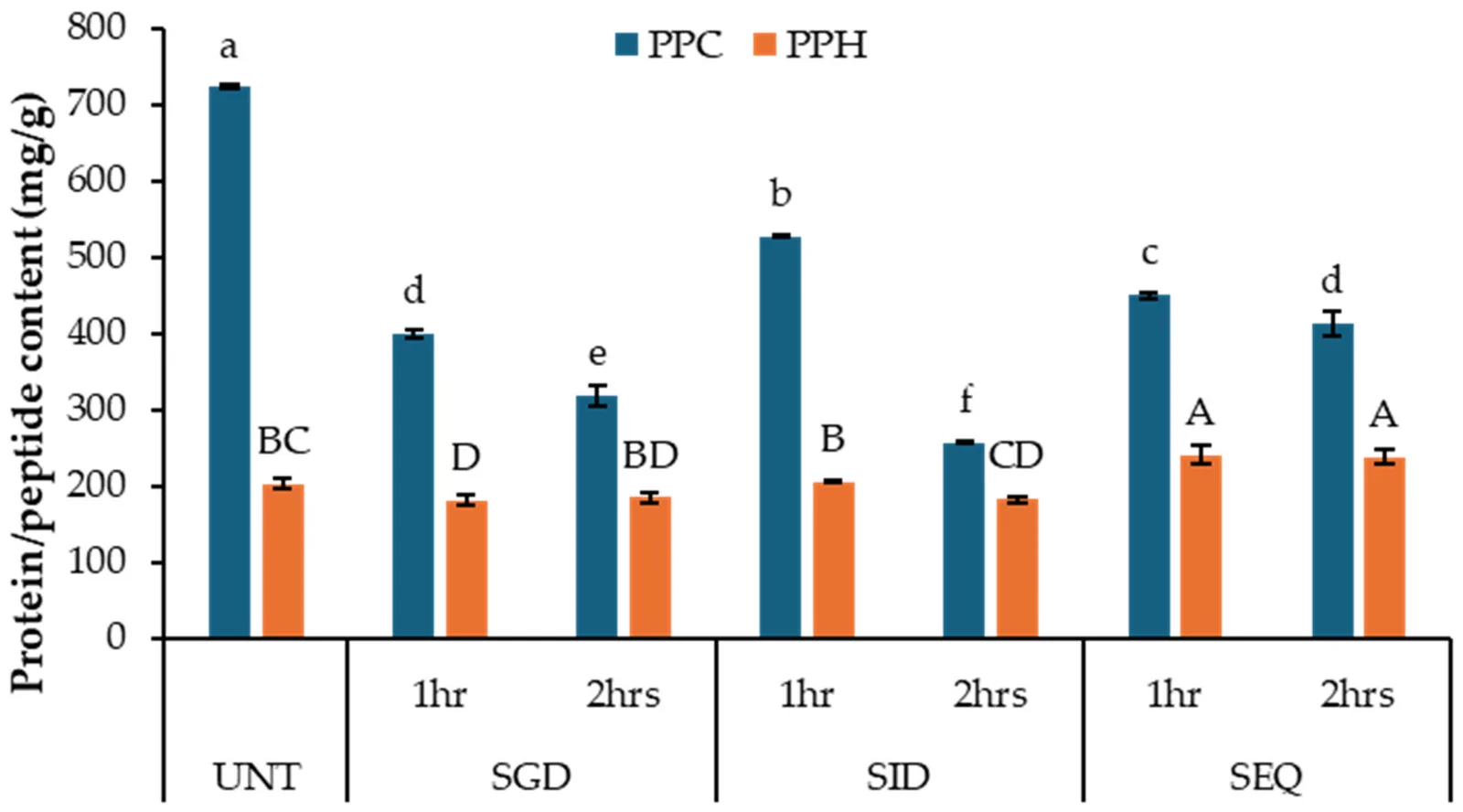
Soluble protein/peptide contents in PPC and PPH before and after simulated GI digestion. Samples: UNT (undigested), SGD (gastric phase), SID (intestinal phase), and SEQ (sequential digestion phase). Different lowercase (PPC) or uppercase (PPH) letters indicate significant differences among treatments (*p* < 0.05).

The effects of SGD, SID and sequential digestion (SEQ) on the protein/peptide content of PPC and PPH are shown in Figure 2. In PPC, all simulated digestion treatments significantly reduced the BCA-detectable protein/peptide content relative to the undigested control (*p* < 0.05) (Figure 2). Gastric and intestinal digestions produced progressive declines of protein/peptide content with increasing digestion time, whereas the effect of digestion time on the protein/peptide concentration in sequential digestion was smaller compared with that of SGD and SID. These trends were consistent with the SDS-PAGE profiles (Figure 1A,B), which showed progressive loss of high-molecular-weight protein bands.

In contrast, the BCA-detectable protein/peptide content of PPH was relatively stable following single-enzyme digestion (SGD or SID), indicating that the peptides generated during the Alcalase hydrolysis were relatively resistant to further proteolysis by either gastric or intestinal enzymes. The SDS-PAGE profiles supported this observation, showing only minor changes in band intensity after single-enzyme digestion (Figure 1C,D). Sequential digestion produced the highest apparent protein/peptide content in PPH, with no additional increase from 1 h to 2 h (Figure 2). This response likely reflects the complementary specificities of pepsin and pancreatin, which generate a greater proportion of low-molecular-weight peptides. Because the BCA assay response depends on peptide size and amino acid composition, but is calibrated using a single protein standard (BSA), these smaller peptides may produce a stronger colorimetric response than the larger peptides present in the undigested hydrolysate, resulting in a higher apparent protein/peptide content rather than an increase in total peptide mass. This interpretation is supported by the substantial loss of SDS-PAGE band intensity following sequential digestion, with only the ~7.7 kDa peptide remaining readily detectable.

**Figure 3:**
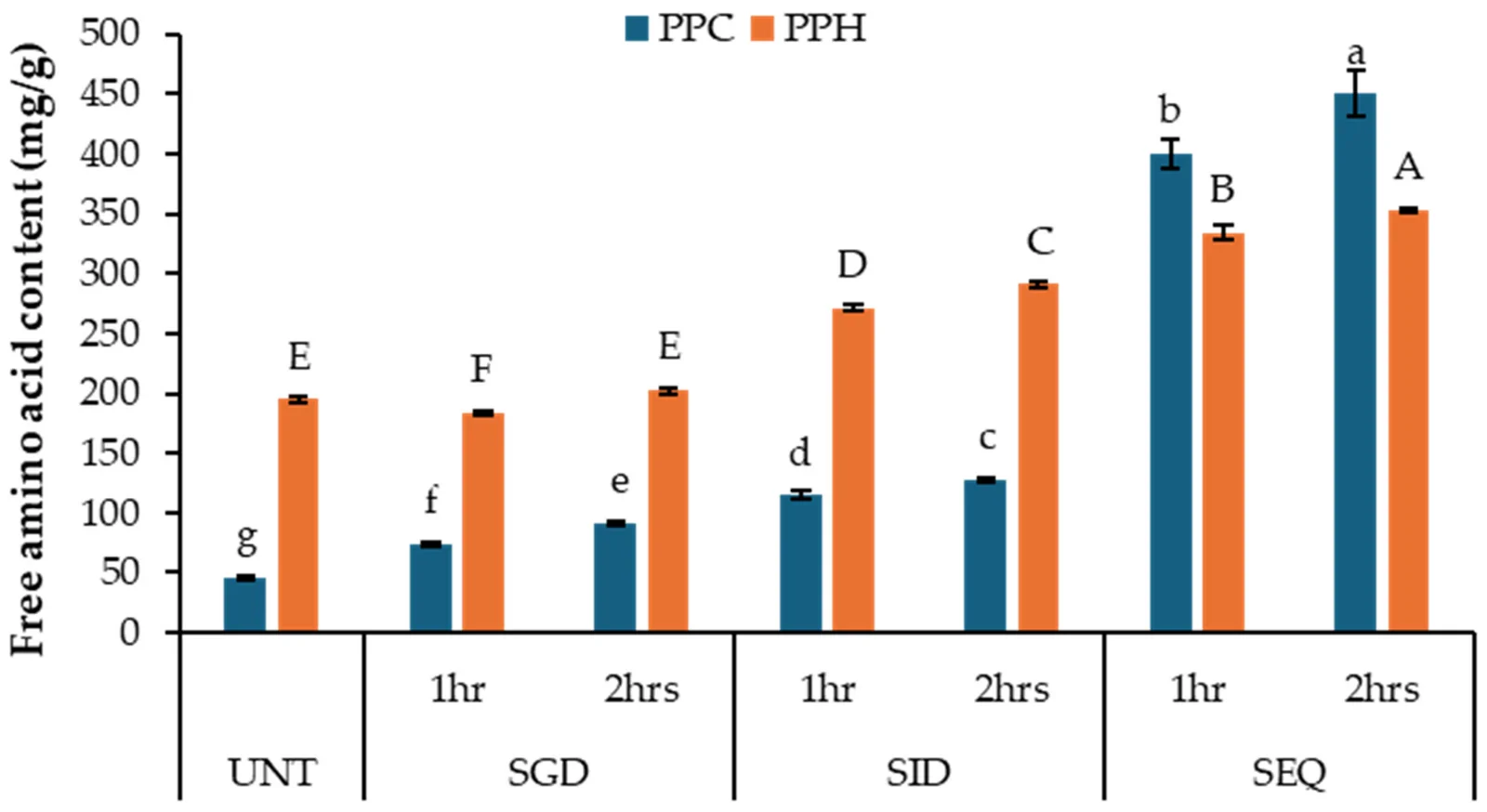
Changes in free amino acid contents in PPC and PPH after simulated GI digestion. Samples: UNT (undigested), SGD (gastric phase), SID (intestinal phase), and SEQ (sequential digestion phase). Different lowercase (PPC) or uppercase (PPH) letters indicate significant differences among treatments (*p* < 0.05).

Free amino acid (FAA) content exhibited the opposite trend to BCA-detectable protein/peptide content, increasing following Alcalase hydrolysis and subsequent simulated gastrointestinal digestion (Figure 3). This inverse relationship reflects progressive protein hydrolysis, whereby peptide cleavage ultimately releases free amino acids. The FAA content in PPC was 45.58 mg/g, greatly lower than that in PPH (195 mg/g) (*p* < 0.05), confirming that Alcalase pretreatment had liberated a substantial pool of free amino acids prior to simulated digestion.

Both SGD and SID significantly increased the FAA content of PPC, and longer digestion time led to greater FAA release. However, SID resulted in higher FAA concentration than SGD at the same digestion time, indicating that SID more effectively cleaves peptide bonds at either the C-terminus or N-terminus of proteins or larger peptides. Notably, the pronounced reduction in BCA-detectable protein/peptide content in pancreatin-digested PPC (Figure 2) was accompanied by only a modest increase in FAA (Figure 3). This apparent disparity suggests that a proportion of the hydrolysis products might be dipeptides rather than FAA. The BCA assay requires tripeptides or larger peptides to form a complex with copper, so dipeptides go undetected [50]. Dipeptides also produce a lower ninhydrin response than free amino acids due to the reduced availability of free α-amino groups for chromophore formation [51]. Sequential digestion produced the highest FAA content, reflecting the complementary hydrolytic activities of pepsin and pancreatin, which promoted more extensive protein degradation than either enzyme alone. The FAA content of sequentially digested PPC also exceeded the sum of the values after gastric and intestinal digestion alone (Figure 3). This may reflect the synergistic action of pepsin and pancreatin when they act in sequence. Pepsin cleavage creates new peptide termini from which the carboxypeptidases in pancreatin release amino acids [30]. Sequential digestion therefore converted a larger share of PPC into FAA and short peptides than either phase alone. Nevertheless, the SGD of PPH did not increase FAA concentration, and the increase in FAA due to sequential digestion was smaller for PPH than for PPC, further confirming that the peptides generated during Alcalase hydrolysis were relatively resistant to further gastrointestinal digestion.

### 3.3. Effect of Simulated GI Digestion on ACE-Inhibitory Activity of PPC and PPH

ACE-inhibitory activity, expressed as IC_50_ values, varied substantially across substrates and enzyme treatments (Table 1). Captopril, the positive control, had an IC_50_ of 5 × 10^−6^ mg/mL, which falls within the manufacturer-reported range of 5.0 × 10^−6^ to 7.6 × 10^−6^ mg/mL, validating the assay conditions. Undigested PPC had the weakest ACE-inhibitory activity, reflecting fewer small peptides capable of effectively inhibiting ACE. The PPH, by contrast, was approximately six-fold more potent (Table 1), showing that Alcalase pre-hydrolysis liberated antihypertensive peptide sequences from intact peanut protein. Peptides with shorter chain lengths and lower molecular weights are more effective ACE inhibitors because they can fit into ACE’s active-site channel, which excludes larger peptides due to spatial constraints [15]. This inverse relationship between molecular weight and ACE-inhibitory activity has been demonstrated across various protein hydrolysates, including soy protein [52], French bean protein [53], and peanut protein [27]. Two-way ANOVA showed a significant interaction between enzyme concentration and digestion time for pepsin-digested PPH, but not for pancreatin-digested PPH, and enzyme type and digestion time did not interact for PPC.

At 1.0 mg/mL, all digestion treatments reduced the IC_50_ of PPC to less than one-third of that of the undigested control, with sequential digestion resulting in the lowest IC_50_ values (highest ACE-inhibitory activity) (Table 1). Two-way ANOVA showed a significant effect of enzyme type (*p* = 0.005) but not digestion time, and no enzyme × time interaction, indicating that the type of protease, not digestion time, determined the ACE-inhibitory activity of digested PPC under the experimental conditions of this study. The initial one-hour digestion lowered the IC_50_ from about 20 mg/mL to between 4.7 and 6.1 mg/mL, but ACE-inhibitory activity plateaued thereafter, suggesting that available peptide bonds for pepsin or pancreatin might be degraded during the first hour of SGD or SID. The modestly lower IC_50_ of sequentially digested PPC relative to single-phase digestion treatments may reflect the complementary cleavage specificity of pepsin and pancreatin, which together access a broader range of peptide bonds and generate sequences not achievable by either enzyme alone.

The IC_50_ of PPH depended on enzyme type, enzyme concentration, and digestion time (Table 1). For SGD, the IC_50_ of PPH after 1 h was similar to that of undigested PPH at pepsin concentrations of 0.5 and 1 mg/mL, but the IC_50_ of PPH decreased significantly when the digestion time was extended to 2 h (*p* < 0.05). Two-way ANOVA showed a significant interaction between pepsin concentration and digestion time (*p* = 0.012). At the same pepsin concentration, the IC_50_ decreased significantly with increasing digestion time (*p* < 0.001). The effect of pepsin concentration on IC_50_ was greater at 2 h than at 1 h. Increasing enzyme concentration from 1 mg/mL to 2 mg/mL did not further improve activity at 2 h, suggesting a plateau beyond which additional pepsin either fails to expose new ACE-inhibitory peptides or begins to degrade existing ones (Table 1). The lower IC_50_ after 2 h of pepsin digestion likely reflects ongoing hydrolysis and the release of low-molecular-weight peptides. Also, pepsin preferentially cleaves bonds involving aromatic and hydrophobic residues, phenylalanine, leucine, and tryptophan, at the C-terminus [30], and peptides carrying Trp, Phe, or Tyr at this position bind ACE 1 more strongly [32]. Pepsin hydrolysis of PPH may therefore favor peptides with C-terminal features associated with enhanced ACE inhibition.

During SID, two-way ANOVA showed significant main effects of pancreatin concentration (*p* < 0.001) and digestion time (*p* = 0.005) with no significant interaction between the two (*p* = 0.60). A higher pancreatin concentration significantly decreased IC_50_, thereby increasing ACE-inhibitory activity. Extending SID from 1 to 2 h lowered the IC_50_ overall, but the difference was not significant at any single pancreatin concentration (Table 1). At the 1 mg/mL reference concentration, the IC_50_ of 2-h pancreatin-digested PPH was similar to that after 2-h pepsin digestion (Table 1). Compared to pepsin, a single endopeptidase that cleaves at hydrophobic and aromatic residues, pancreatin contains endopeptidases trypsin, α-chymotrypsin, and elastase, as well as exopeptidases carboxypeptidases A and B, which cleave at distinct sites [30]. Trypsin cleaves after Arg and Lys, and α-chymotrypsin after Phe, Tyr, and Trp. Carboxypeptidase A removes aromatic and branched aliphatic residues from the C-terminus, and carboxypeptidase B removes Arg and Lys, once these residues are exposed by the endopeptidases [30]. The continued reduction in IC_50_ with increasing pancreatin concentration may therefore reflect the broader range of peptide sequences generated by the protease mixture, which cleave at different sites and produce peptides with different sequences, lengths, and conformations, each potentially interacting with ACE through competitive, non-competitive, or mixed inhibitory mechanisms [32].

Sequential digestion achieved the most extensive hydrolysis of PPH, as shown by SDS-PAGE (Figure 1C,D), but did not yield the lowest IC_50_ values. The 1-h sequentially digested PPH had the highest mean IC_50_ among the PPH treatments (Table 1), but neither sequential digestion time changed the IC_50_ significantly from that of undigested PPH. This apparent contradiction between hydrolysis extent and bioactivity likely reflects a balance between the release of new inhibitory peptides and the degradation of existing ones. A smaller average peptide size improves ACE inhibition only when the peptides keep the sequence features linked to better ACE inhibitory activity, such as Trp, Phe, or Tyr at the C-terminus [32]. Pepsin first cleaves larger peptides in PPH, which can release inhibitory sequences. The pancreatin that follows contains carboxypeptidase A, which removes C-terminal aromatic residues [30], and endopeptidases that can cut existing inhibitory peptides into inactive fragments. The higher free amino acid content of sequentially digested PPH (Figure 3) indicates that the exopeptidases in pancreatin broke part of the peptide pool down to FAA rather than into new inhibitory peptides. A similar pattern was reported for soy protein, where pepsin digesta lost ACE-inhibitory activity when it was further digested with pancreatin [54]. Lower molecular weight therefore favors ACE inhibition only until peptides are hydrolyzed to free amino acids. Single-phase digestion maintained or improved the ACE-inhibitory activity of PPH, while sequential digestion did not change it significantly (Table 1).

### 3.4. Effect of Serum Incubation on ACE-Inhibitory Activity of PPH

**Figure 4:**
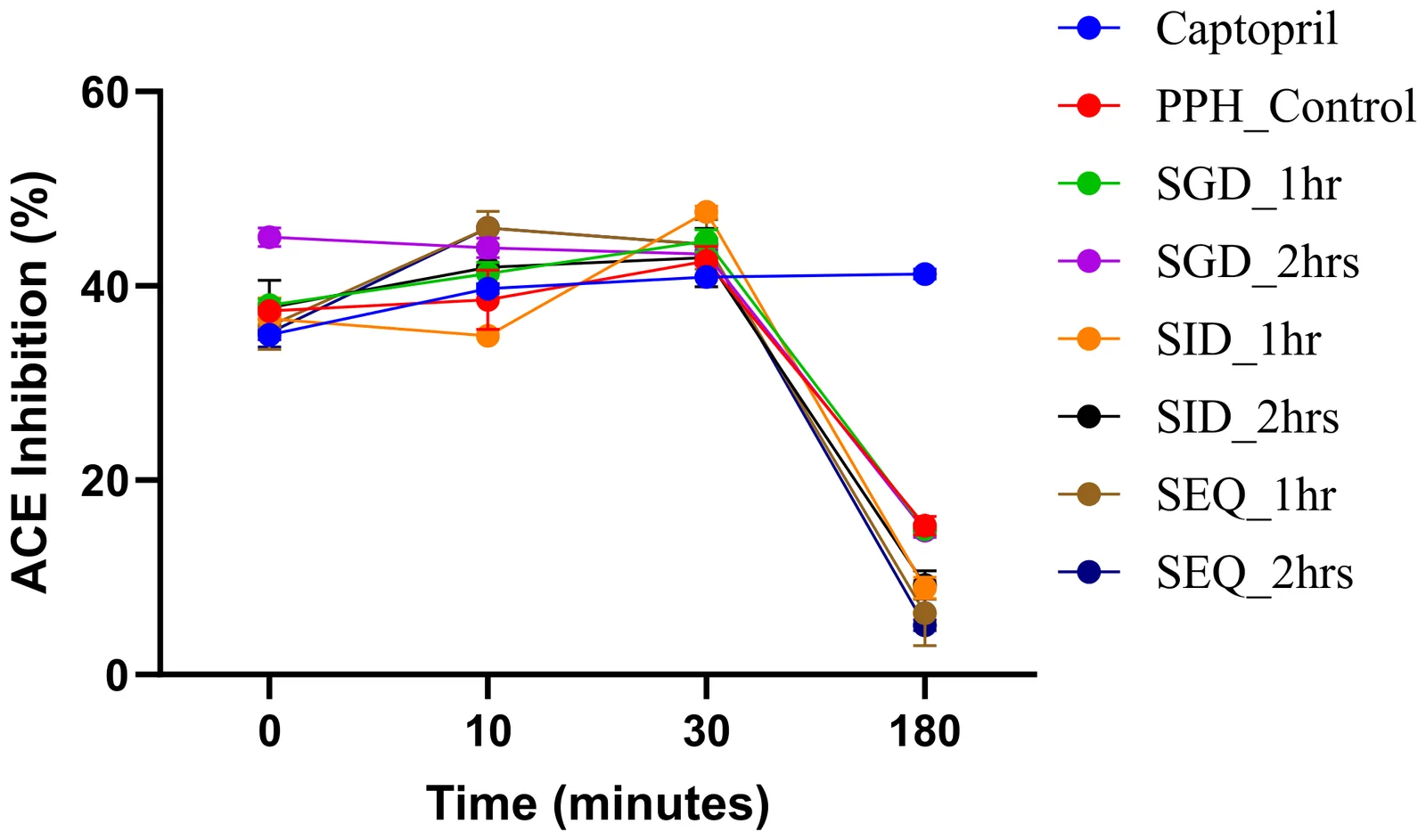
ACE inhibitory activity (%) of undigested PPH and simulated gastric (SGD), intestinal (SID), or sequential (SEQ) digested PPH samples after incubation in human serum for 0, 10, 30, and 180 min, measured at 2 mg/mL. Captopril (5 µg/mL) was the positive control. Values are mean ± SD (*n* = 3). Differences among incubation times within each sample were tested by two-way ANOVA followed by Tukey’s test (*p* < 0.05). Means ± SD with significance letters are given in Table S1.

Following absorption into the bloodstream, the peptides must withstand serum peptidase hydrolysis to reach their target organs intact and exert their antihypertensive effect; alternatively, less potent, larger ACE-inhibitory peptides may be hydrolyzed by serum peptidases to form more potent ACE inhibitors. In this study, PPH and simulated digested PPH samples were incubated in human serum for 10, 30, and 180 min. ACE inhibition did not decline in the first 30 min and was significantly higher at 10 or 30 min than at 0 min in most samples (Figure 4, Table S1), indicating that the peptides either survived serum peptidases or were converted into peptides that remained active within the physiologically relevant window of circulation. This is significant because therapeutic peptides are typically cleared from the bloodstream within 2–30 min through plasma peptidase degradation and renal elimination [55]. Captopril also showed a similar early increase. Captopril is not changed by serum peptidases, so part of the rise in the PPH samples may come from the assay rather than from new inhibitory peptides. By 3 h, however, all PPH samples showed a substantial loss of ACE-inhibitory activity (*p* < 0.05 against all earlier times): inhibition fell below 16% in SGD samples and below 10% in both SID- and sequentially digested samples. This decline is expected, as the 3-h period extends well beyond the circulating half-life of bioactive peptides. Captopril, by contrast, showed no loss of ACE inhibition during the incubation period, consistent with its known resistance to enzymatic degradation as a synthetic peptidomimetic [56].

These results describe how PPH peptides behave once they reach the bloodstream, and intestinal absorption was not evaluated in this study. Di- and tripeptides released by extensive hydrolysis can be absorbed intact through peptide transporter 1 (PepT1), and larger peptides can cross by paracellular transport or transcytosis, depending on their size, flexibility, and hydrophobicity [15]. Brush-border peptidases may also cleave larger peptides into shorter, absorbable fragments [15]. Part of the ACE-inhibitory activity of PPH may therefore reach the circulation, but the amount that does so in vivo remains to be measured. Protective strategies such as emulsion-based encapsulation and liposome technology have been shown to enhance the delivery of intact bioactive peptides to the intestinal site of absorption [57,58,59]. By increasing the concentration of intact peptides available for epithelial uptake, these delivery strategies may enhance the proportion of bioactive peptides reaching the systemic circulation, thereby sustaining the ACE-inhibitory activity of PPH within the critical window observed in serum.

### 3.5. Effect of PPH on Blood Pressure in Spontaneously Hypertensive Rats

**Figure 5:**
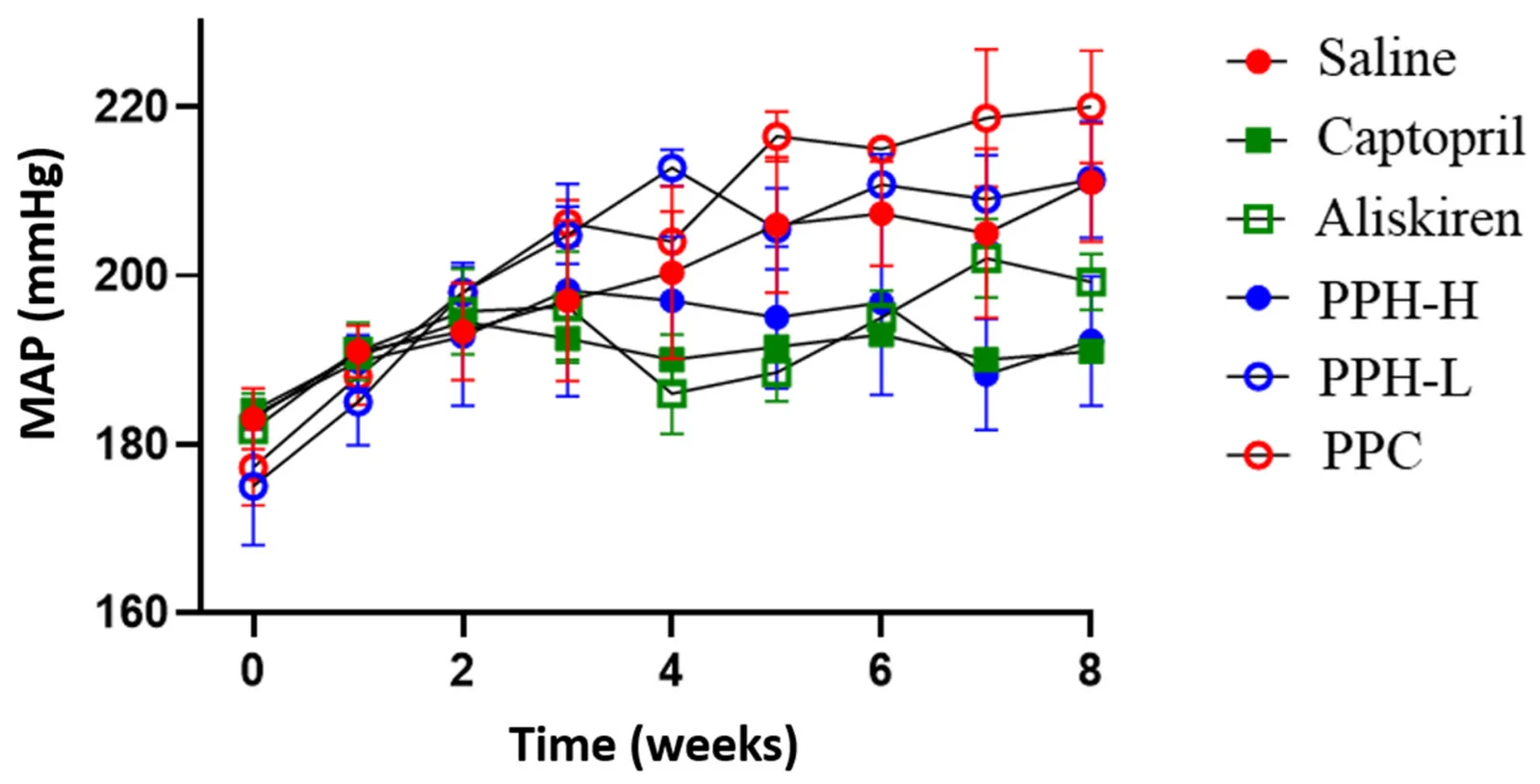
Mean arterial pressure (MAP, mmHg) of spontaneously hypertensive rats in different treatment groups (*n* = 8) over 8 weeks following daily oral administration of saline, captopril, aliskiren, PPH at high dose (PPH-H), PPH at low dose (PPH-L), and PPC. Values are mean ± SD. Differences within each week were assessed by Tukey’s test (*p* < 0.05). The MAP means ± SD with significance letters are given in Table S2.

The effect of oral intake of PPH on blood pressure was assessed by the degree to which it attenuated the MAP of SHRs over an 8-week study period (Figure 5). In all treatment groups, the MAP of SHRs increased in the first 3 weeks. The MAP of rats in the low-dose PPH group (250 mg/kg bw/day), the saline group, and the PPC group kept rising or stayed high after week 3. MAP in the captopril group fell slightly after week 2 and stayed stable from week 3 to week 8. MAP in the aliskiren group was lowest at week 4 and then rose slightly thereafter. MAP in the high-dose PPH group (1000 mg/kg bw/day) decreased after week 3, reached its lowest value at week 7, and stayed low at week 8. From week 3 to week 6, the MAP of rats in Captopril, Aliskiren and high-dose PPH groups were slightly different from each other (*p* ≤ 0.05) but significantly lower than that of other groups (*p* < 0.05). Among these three groups, the MAP of rats in Captopril and high-dose PPH groups were not significantly different but significantly lower than those in Aliskiren groups from week 7-week 8 (*p* < 0.05) (Table S2).

The effect of PPH on MAP was dose-dependent. The low-dose had the same effect as the saline control and did not show blood-pressure lowering effect, while the high-dose PPH showed blood pressure-lowing effect comparable to captopril and marginally better than those of Aliskiren in this model (*p* ≤ 0.05) (Figure 5). This indicates that 250 mg/kg did not deliver enough ACE-inhibitory peptides to attenuate the rise in MAP, whereas the greater amount of peptide delivered at 1000 mg/kg produced a measurable effect.

By the end of the study, the high dose (1000 mg/kg bw) of PPH had attenuated the rise in MAP. Body surface area conversion [60] gives a human equivalent dose of about 160 mg/kg bw/day for the effective rat dose, or about 9.7 g/day for a 60 kg adult, but the efficacy and safety of this dose in humans remain to be tested. However, PPC resulted in the highest MAP of all groups, higher than even the saline control (Figure 5). This confirms that the pre-enzymatic hydrolysis with Alcalase is critical in releasing antihypertensive peptides from peanut protein.

At week 8, MAP in the PPC group was higher than that in the saline group (*p* < 0.05). One possible explanation involves peanut agglutinin (PNA), a peanut lectin that resists digestion and can reach the blood intact after peanut intake [61]. PNA induces endothelial cells to secrete the inflammatory cytokines interleukin-6 (IL-6) and monocyte chemoattractant protein-1 (MCP-1) [62]. SHRs already have higher plasma IL-6 than normotensive rats [63], renal IL-6 rises as their hypertension develops [64], and inflammation contributes to hypertension in SHRs [65]. SHRs also show increased intestinal permeability [66,67], which may allow more intact dietary protein to be absorbed. A vascular effect of peanut lectin has been reported before in animals, as lowering the lectin content of peanut oil reduced its atherogenicity in cholesterol-fed rabbits [68]. Alcalase hydrolysis would be expected to degrade PNA in PPH, which may explain why PPH did not have this effect. Intestinal absorption of PPH peptides was not measured, so the link between their serum stability and the blood pressure response remains to be confirmed. MAP was measured only 4 h after gavage, so the design does not separate an acute effect of each dose from a chronic effect of repeated dosing.

### 3.6. Effect of Simulated GI Digestion on Relative Secondary Structure of PPC and PPH

**Figure 6:**
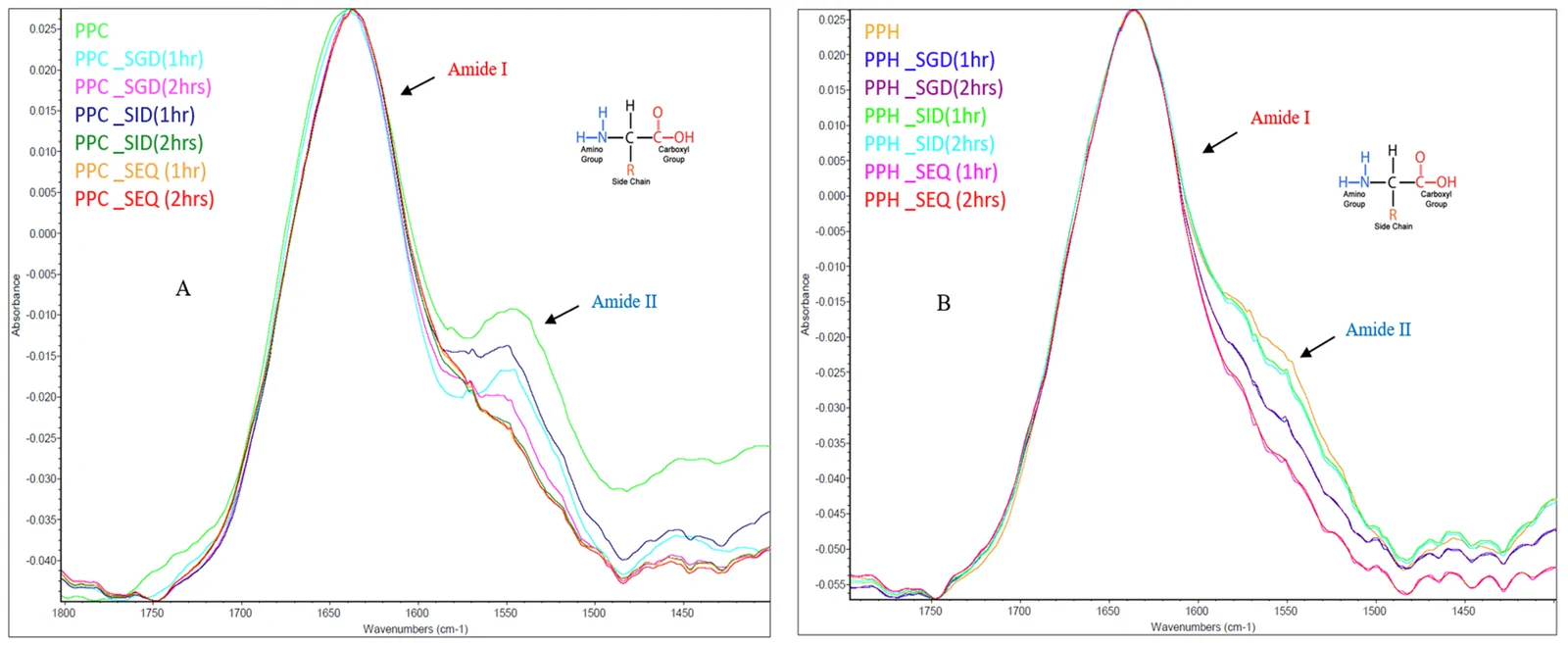
Superimposed FTIR spectra of (**A**) PPC and (**B**) PPH following simulated GI digestion under SGD (gastric), SID (intestinal), or SEQ (sequential) conditions.

The FTIR spectra of PPC and PPH are shown in Figure 6A,B. The amide I band (1600–1700 cm^−1^) arises from C=O stretching of the peptide backbone, while the amide II band (1510–1580 cm^−1^) arises from N-H bending and C-N stretching of the peptide bond [69]. In the Amide I region, PPC showed a strong, well-defined peak in its native state, although changes in peak shape and broadening indicated shifts in secondary-structure composition. PPH showed even smaller changes in the Amide I region relative to PPC. In the Amide II region, both proteins showed more pronounced changes upon digestion, confirming peptide bond cleavage. For both PPC and PPH, sequential digestion produced the lowest Amide II intensity, consistent with the extensive peptide bond cleavage seen on SDS-PAGE (Figure 1C,D).

**Figure 7:**
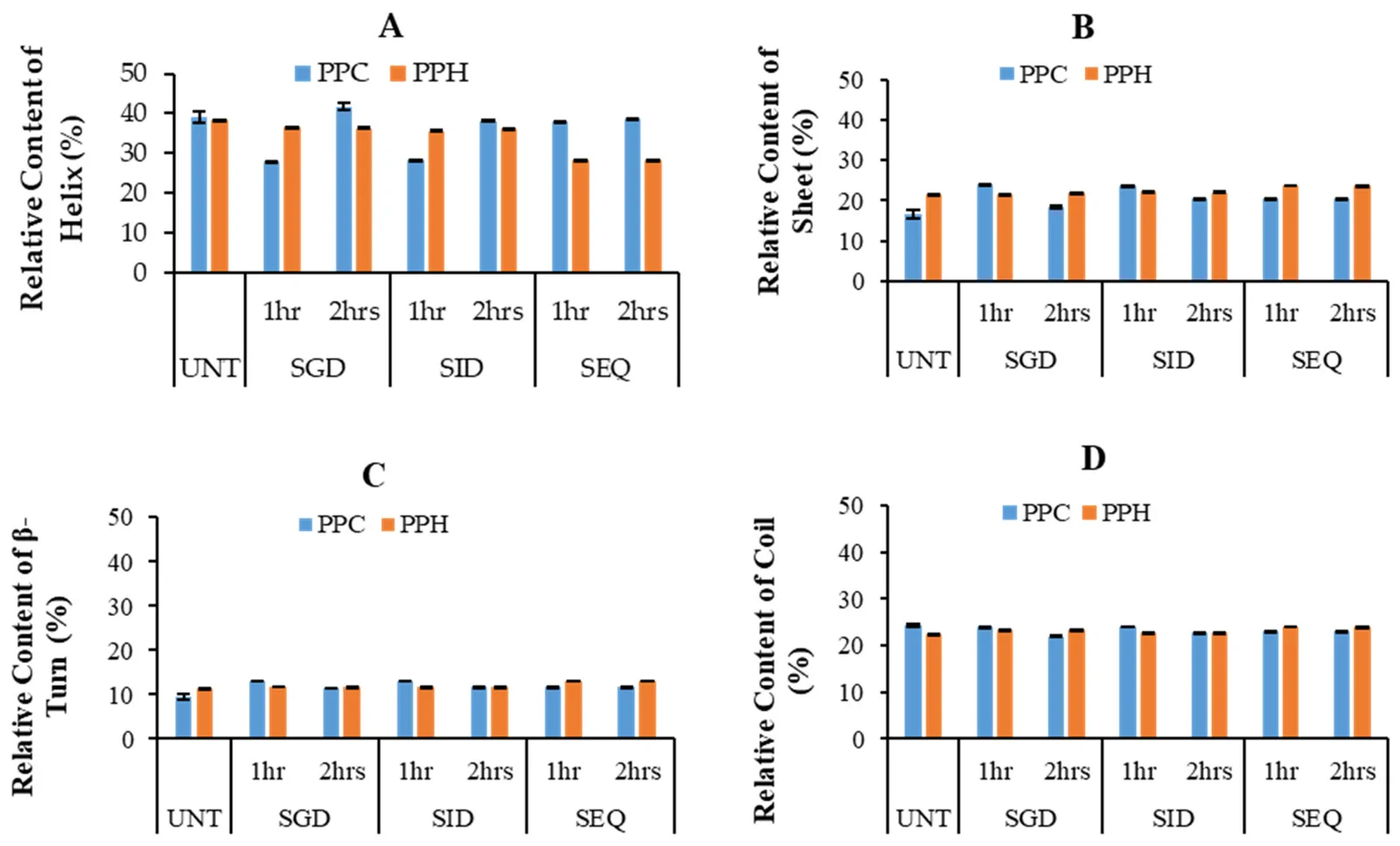
Relative secondary-structure composition of PPC and PPH as affected by simulated GI digestion under SGD (gastric), SID (intestinal), or SEQ (sequential) conditions: (**A**) α-helix, (**B**) β-sheet, (**C**) β-turn, and (**D**) random coil. Values are mean ± SD (*n* = 3).

α-Helix was the predominant secondary structure in undigested PPC and PPH (Figure 7A). This agrees with the FTIR spectra, in which the amide I band was centered near 1655 cm^−1^ (Figure 6A,B), a region characteristic of α-helical structures, whereas β-sheet structures typically absorb closer to 1630 cm^−1^ [70]. Alcalase hydrolysis increased the proportion of β-sheet structures in PPH, although α-helix remained the predominant secondary structure, despite extensive proteolysis to low-molecular-weight peptides. A similar shift toward increased β-sheet content following enzymatic hydrolysis has been reported for defatted peanut flour treated with a multi-protease preparation [71].

SGD of PPC for 1 h caused a decrease in α-helix content, accompanied by a concurrent increase in β-sheet and β-turn, indicating initial disruption of helical regions (Figure 7A–C). After 2 h, α-helix content rebounded to near-original levels, while β-sheet content decreased, suggesting a non-linear structural rearrangement that may involve hydrolysis of different structural domains at different rates [72] or secondary-structure transitions between α-helix and β-sheet conformations [73]. In PPH, SGD produced only minor changes in secondary structure regardless of digestion time.

The effects of SID on the secondary structural elements in PPC were similar to those of SGD, with reduced α-helix and increased β-sheet in the first hour, followed by partial recovery of α-helix after 2 h of digestion. In PPH, pancreatin also produced minimal structural changes, further supporting the resistance of the pre-hydrolyzed protein to pepsin or pancreatin (Figure 7A,B). This contrasting behavior between PPC and PPH likely reflects differences in the availability of peptide bonds for attack by digestive enzymes. Many peptide bonds in native proteins are initially inaccessible to enzymes because they are located within the protein globule, requiring disruption of the protein structure to expose them for successful enzymatic cleavage [74].

Sequential digestion of PPC caused little change in α-helix, an increase in β-sheet and β-turn, and a slight decrease in random coil compared to the undigested PPC. The sequential digestion of PPH decreased α-helix content, but increased β-sheet, β-turn, and random coil contents. For PPH, the content of each secondary structure was similar after 1 h and 2 h of digestion under each treatment, suggesting that structural changes occurred rapidly within the first hour. The relative stability of these structural components in PPH across enzymatic treatments suggests that β-turn and random-coil conformations in PPH may be less susceptible to GI enzymes, as different proteases have distinct preferences for secondary-structure targets [75] (Figure 7C,D).

### 3.7. Correlation Between Secondary Structure and ACE-Inhibitory Activity

The correlation coefficients between the relative contents of each secondary structure and ACE-inhibitory activity are shown in Table 2. In PPC digesta, there were negative correlations between β-sheet (r = −0.66)/β-turn (r = −0.78) contents and IC_50_, so ACE inhibition tended to increase with the increments of relative contents of these structures. Only the β-turn-IC_50_ correlation was significant at *p* < 0.05, and the β-sheet—IC50 correlation was significant at *p* < 0.10. Increasing α-helix and random coil contents in PPC correlated with higher IC_50_ (Table 2), indicating lower ACE inhibition, but neither correlation was significant. β-sheet conformations of casein-derived peptides have been shown to be favorable for ACE binding [38].

PPH showed opposing trends. Extensive hydrolysis by Alcalase had already released most peptides in PPH from their native conformations before GI digestion, and short sequences rarely keep a defined fold once released from the parent protein [33]. The α-helix showed a moderate negative correlation with IC_50_ in PPH (r = −0.56) (Table 2), although this correlation was not significant (*p* = 0.19). Given that the change in the helical content was only modest across pepsin and pancreatin treatments, these resistant helical peptides may partly account for the observed ACE-inhibitory activity. This is consistent with the sequential digestion results, in which the loss of α-helix coincided with a small, non-significant rise in IC_50_ after 1 h (Table 1). The relationship between the α-helix and ACE inhibition, however, has been inconsistent across studies. Yu et al. (2011) [39] suggested that the α-helix conformation observed in the egg white peptide QIGLF might associate with its ACE-inhibitory activity, while Cian et al. (2015) [37] observed the opposite trend in wheat gluten hydrolysate, where higher α-helix content correlated with weaker ACE inhibition. Together with the moderate correlation observed in the present study, these contradicting findings suggest that the role of secondary structure in ACE inhibition is protein- and peptide-specific rather than universal.

Furthermore, the extensive hydrolysis by Alcalase likely produced shorter peptides in PPH, for which the primary sequence rather than the secondary structure is expected to be the dominant factor in ACE binding [32]. This may partly explain why the correlations between secondary structure and IC_50_ in PPH were only moderate, since many of the peptides may be too short to hold a stable secondary structure [33]. Higher β-sheet and β-turn content in PPH nonetheless correlated with reduced ACE-inhibitory activity (Table 2), possibly due to peptide aggregation that produced conformations that hindered access to the ACE active site. Peptide aggregation has been reported in β-sheet-rich peanut agglutinin [76].

Identifying the peptides responsible for ACE inhibition is the next step of this work. Peptidomic analysis of PPH, its GI digesta, and its serum digesta by liquid chromatography-tandem mass spectrometry (LC-MS/MS) would show which sequences survive each stage of digestion and would allow a direct test of the relationship between peptide structure and activity.

## 4. Conclusions

This study revealed that PPH produced by Alcalase hydrolysis of PPC had about six-fold greater ACE-inhibitory activity than PPC, confirming that enzymatic hydrolysis liberates antihypertensive peptides from peanut protein. PPH was more resistant to gastrointestinal digestion than PPC, and single-phase digestion maintained or increased its ACE-inhibitory activity while sequential digestion did not change it significantly, suggesting that although some additional bioactive peptide generation occurred during digestion, the ACE-inhibitory activity of PPH may be largely attributable to Alcalase-generated peptides that remained partially resistant to gastrointestinal degradation. Correlation analysis suggested that the bioactivity of PPH depends more on peptide properties, such as sequence, molecular weight, and hydrophobicity, than on secondary structure. PPH peptides maintained their ACE-inhibitory activity in human serum within the physiologically relevant circulation window of 10–30 min, so they may remain active after absorption. Most of this activity was lost by 180 min. PPH at a daily dose of 1 g/kg bw attenuated the rise in MAP of SHRs, while the same dose of PPC had no effect, which suggests that hydrolysis with Alcalase was needed to release the antihypertensive components of peanut protein. ACE-inhibitory peptides from PPH that can reach the circulation may have contributed to this effect, but intestinal absorption was not measured, and this link remains to be confirmed. Protective delivery strategies, such as encapsulation, may be needed to maximize the proportion of intact bioactive peptides that reach systemic circulation. Consequently, PPH holds promise as a nutraceutical or functional food ingredient for blood pressure management while adding value to defatted peanut residue, a by-product of peanut oil extraction. Further studies are needed to establish the therapeutic potential of PPH or PPH-enriched foods, including identification of the bioactive peptide sequences by peptidomic analysis, measurement of their intestinal absorption, and human clinical trials of safety and efficacy.

## Figures and Tables

**Table 1:** IC_50_ values (mg/mL) of PPC and PPH digested at different enzyme concentrations and digestion times.

Protein	Treatment	Enzyme Concentration (mg/mL)	1 h Digestion	2 h Digestion
Captopril	-	-	5 × 10^−6^ ± 4 × 10^−7^	
PPC	-	-	20.27 ± 3.27 ^a^	
	Pepsin (SGD)	1	5.56 ± 0.83 ^b^	5.65 ± 0.19 ^b^
	Pancreatin (SID)	1	6.14 ± 0.77 ^b^	5.57 ± 0.36 ^b^
	Sequential digestion	1 (pepsin) → 1 (pancreatin)	4.71 ± 0.26 ^b^	4.66 ± 0.27 ^b^
PPH	-	-	3.58 ± 0.05 ^ab^	
	Pepsin (SGD)	0.5	3.61 ± 0.17 ^ab^	3.03 ± 0.25 ^bcd^
		1	3.63 ± 0.06 ^ab^	2.55 ± 0.11 ^cg^
		1.5	3.27 ± 0.12 ^bc^	2.30 ± 0.07 ^dg^
		2	3.03 ± 0.21 ^bcd^	2.51 ± 0.07 ^cg^
	Pancreatin (SID)	0.5	3.43 ± 0.36 ^ab^	3.11 ± 0.34 ^bc^
		1	3.30 ± 0.12 ^bc^	2.90 ± 0.32 ^bcde^
		1.5	2.87 ± 0.18 ^cdef^	2.08 ± 0.43 ^fg^
		2	2.11 ± 0.31 ^eg^	1.78 ± 0.52 ^g^
	Sequential digestion	1 (pepsin) → 1 (pancreatin)	4.12 ± 0.26 ^a^	3.62 ± 0.19 ^ab^

Values are means ± SD (*n* = 3). Within each protein (PPC or PPH), means with different lowercase letters differ significantly (*p* < 0.05). The 1 mg/mL concentration is the reference enzyme concentration used in the simulated GI model. IC_50_ = the concentration that inhibits 50% of ACE activity.

**Table 2:** Pearson correlation coefficients (*r*) between secondary structure content and ACE-inhibitory activity (IC_50_) for PPC and PPH, with two-tailed *p*-values in parentheses.

Secondary Structure	r for PPC	r for PPH
α-Helix	0.23 (0.61)	−0.56 (0.19)
β-sheet	−0.66 (0.10)	0.52 (0.23)
β-turn	−0.78 (0.040)	0.59 (0.16)
Random coil	0.53 (0.22)	0.41 (0.36)

Negative values indicate that a higher proportion of the structural element is associated with a lower IC_50_, that is, stronger inhibition. Two-tailed *p*-values are given in parentheses (*n* = 7 samples per substrate).

## Data Availability

The raw data supporting the conclusions of this article will be made available by the authors on request.

## Supplementary Materials

The following supporting information can be downloaded at: https://www.mdpi.com/article/10.3390/foods15213908/s1, Table S1: ACE-inhibitory activity (%) of undigested and simulated digested PPH after incubation in human serum; Table S2: Mean arterial pressure (MAP, mmHg) of spontaneously hypertensive rats in each treatment group before oral gavage (week 0) and each week over an 8-week daily oral gavage.

## Abbreviations

The following abbreviations are used in this manuscript:

ACE	Angiotensin I-converting enzyme
ATR	Attenuated total reflectance
BCA	Bicinchoninic acid
BSA	Bovine serum albumin
FAA	Free amino acid
FAPGG	N-[3-(2-furyl)acryloyl]-Phe-Gly-Gly (ACE substrate)
FT-IR	Fourier transform infrared spectroscopy
GI	Gastrointestinal
IACUC	Institutional Animal Care and Use Committee
IC_50_	Half-maximal inhibitory concentration
IL-6	Interleukin-6
LC-MS/MS	Liquid chromatography-tandem mass spectrometry
MAP	Mean arterial pressure
MCP-1	Monocyte chemoattractant protein-1
MCT	Mercury cadmium telluride
MW	Molecular weight
NO	Nitric oxide
PepT1	Peptide transporter 1
PNA	Peanut agglutinin
PPC	Peanut protein concentrate
PPH	Peanut protein hydrolysate
RAS	Renin-angiotensin system
SBP	Systolic blood pressure
SDS-PAGE	Sodium dodecyl sulfate-polyacrylamide gel electrophoresis
SEQ	Sequential digestion
SGD	Simulated gastric digestion
SGF	Simulated gastric fluid
SHR	Spontaneously hypertensive rat
SID	Simulated intestinal digestion
SIF	Simulated intestinal fluid
