# Supplementary material for: Antihypertensive Potential and Bioavailability of Peanut Protein Hydrolysate Derived from Partially Defatted Peanut Flour
Source: Foods. 2026 Oct 31;15(21):3908. doi: 10.3390/foods15213908 (PMC13762779; doi:10.3390/foods15213908)
Supplement: Supplementary file 1 [file foods-15-03908-s001.zip › foods-4564955-supplementary.pdf]

Supplementary Materials

Antihypertensive Potential and Bioavailability of Peanut Protein Hydrolysate Derived from Partially Defatted Peanut Flour

Table S1. ACE-inhibitory activity (%) of undigested and simulated digested PPH after incubation in human serum.

	Incubation time (min)			
**Sample**	**0**	**10**	**30**	**180**
Undigested PPH	37.42 ± 0.33^{b}	38.57 ± 3.07^{b}	42.60 ± 1.54^{a}	15.32 ± 0.97^{c}
SGD 1 h	38.04 ± 0.66^{b}	41.25 ± 1.16^{b}	44.61 ± 1.16^{a}	15.00 ± 0.56^{c}
SGD 2 h	45.00 ± 1.00^{a}	43.94 ± 1.01^{a}	43.27 ± 1.17^{a}	14.84 ± 0.69^{b}
SID 1 h	36.64 ± 2.14^{b}	34.88 ± 0.00^{b}	47.63 ± 0.58^{a}	8.91 ± 1.11^{c}
SID 2 h	37.75 ± 2.81^{b}	41.92 ± 1.74^{a}	42.93 ± 3.02^{a}	9.23 ± 1.47^{c}
SEQ 1 h	35.87 ± 2.42^{b}	45.95 ± 1.74^{a}	44.27 ± 2.54^{a}	6.34 ± 3.38^{c}
SEQ 2 h	35.20 ± 1.45^{b}	45.95 ± 0.00^{a}	44.27 ± 0.58^{a}	5.06 ± 0.55^{c}
Captopril	34.98 ± 0.52^{b}	39.74 ± 0.51^{a}	40.93 ± 0.00^{a}	41.23 ± 0.52^{a}

PPH samples were tested at 2 mg/mL and captopril (positive control) at 5 µg/mL. Values are mean ± SD (n = 3). Within each sample, means with different superscript letters differ significantly among incubation times (two-way ANOVA followed by Tukey's test, $p < 0.05$).

Table S2. Mean arterial pressure (MAP, mmHg) of spontaneously hypertensive rats in each treatment group before oral gavage (week 0) and each week over an 8-week daily oral gavage.

Week	Treatment Groups					
	**Saline**	**Captopril**	**Aliskiren**	**PPH-H**	**PPH-L**	**PPC**
0	182.9 ± 3.6^{a}	183.9 ± 2.1^{a}	181.7 ± 2.4^{a}	182.9 ± 3.6^{a}	174.9 ± 7.0^{a}	177.2 ± 4.6^{a}
1	190.9 ± 3.1^{a}	191.0 ± 3.5^{a}	190.6 ± 2.8^{a}	190.7 ± 2.2^{a}	185.0 ± 5.2^{a}	187.7 ± 3.1^{a}
2	193.3 ± 5.7^{a}	195.8 ± 5.1^{a}	195.8 ± 5.1^{a}	192.9 ± 8.4^{a}	197.9 ± 3.4^{a}	197.9 ± 3.4^{a}
3	196.9 ± 9.5^{ab}	192.5 ± 2.8^{b}	196.3 ± 6.6^{ab}	198.0 ± 12.6^{ab}	204.7 ± 3.4^{a}	206.6 ± 2.1^{a}
4	200.3 ± 10.2^{a}	190.0 ± 3.0^{b}	186.0 ± 4.9^{b}	196.9 ± 7.6^{ab}	212.7 ± 2.2^{a}	204.0 ± 3.5^{a}
5	206.0 ± 8.0^{ab}	191.3 ± 1.0^{b}	188.4 ± 3.4^{b}	195.0 ± 8.4^{ab}	205.5 ± 4.8^{ab}	216.5 ± 2.8^{a}
6	207.4 ± 6.2^{a}	193.2 ± 0.8^{b}	195.2 ± 3.0^{b}	196.4 ± 10.6^{ab}	210.8 ± 3.6^{a}	214.8 ± 1.0^{a}
7	205.0 ± 10.0^{ab}	190.0 ± 1.3^{c}	202.0 ± 4.6^{ab}	188.1 ± 6.6^{c}	209.0 ± 5.2^{ab}	218.7 ± 8.1^{a}
8	211.0 ± 7.0^{ab}	190.9 ± 0.7^{c}	199.2 ± 3.3^{b}	192.1 ± 7.6^{bc}	211.3 ± 6.9^{ab}	220.0 ± 6.7^{a}

Values are mean ± SD (n = 8 rats per group). PPH-H, high-dose PPH (1,000 mg/kg bw/day); PPH-L, low-dose PPH (250 mg/kg bw/day); PPC was given at 1,000 mg/kg bw/day.

Within each week, means sharing a superscript letter do not differ significantly (two-way repeated measures ANOVA, followed by Tukey’s test, $p < 0.05$).
